# To operate or not to operate? The impact of surgical treatment on quality of life in women with ovarian endometriosis

**DOI:** 10.3389/fgwh.2025.1606768

**Published:** 2025-06-13

**Authors:** Emanuela Spagnolo, David Ramiro-Cortijo, Blanca Díaz Fuentes, María Suarez Vega, Lucía Calvillo-Fernández, Ana López, Alicia Hernández

**Affiliations:** ^1^ Department of Obstetrics and Gynecology, Endometriosis Unit, Hospital Universitario La Paz, Madrid, Spain; ^2^ Hospital La Paz Institute for Health Research (IdiPAZ), Hospital Universitario La Paz, Madrid, Spain; ^3^ Department of Physiology, Faculty of Medicine, Universidad Autónoma de Madrid, Madrid, Spain

**Keywords:** quality of life, deep endometriosis, ovarian endometriosis, endometrioma, surgical treatment

## Abstract

**Background:**

Endometriosis is a chronic gynecological condition that impacts on women's health, reducing their quality of life. Ovarian endometriosis (OE) and deep endometriosis (DE) are the primary manifestations. While surgical intervention in OE is common, its effects on quality of life remain debated. This study aims to assess global health perception and quality of life in women with OE with and without surgery to inform targeted interventions strategies.

**Methods:**

This cross-sectional observational study was conducted at Hospital Universitario La Paz (Spain) and included women aged 25–55 diagnosed with OE, operated (OE-S) or not (OE-NS), as well as those with DE who had surgical resection. Women without endometriosis (control) was also included. Health-related quality of life was measured by SF-36, while pain perception, social support, and endometriosis-specific quality of life were assessed through validated instruments.

**Results:**

Regarding global health, physical and social functions, emotional role, body pain, and global mental health did not find difference between groups. However, women with DE and OE-NS had significantly lower physical role and global health scores compared to controls. Vitality and physical component scores were lower in DE, while pain sensitivity was higher in OE-NS and DE. Social support perception was reduced in women with OE compared to controls. Quality of life was significantly lower in DE and OE-NS groups, with OE-S showing intermediate scores. Psychological well-being and endometriosis-related support were significantly low across all endometriosis groups. Sexual and occupational functions were higher in OE- S than in OE- NS and DE. Reproductive function was impaired in OE- NS compared to controls, while menstrual characteristics were significantly altered in all endometriosis groups. OE- S exhibited intermediate health and quality of life patterns between control and DE groups, whereas OE- NS was more similar to DE.

**Conclusion:**

Psychological well-being and social support are reduced in all endometriosis groups, but surgical treatment in women with ovarian endometriosis preserve vitality, sexual, and occupational functions. A multidisciplinary approach is essential to improve quality of life in women with endometriosis.

## 1 Introduction

Endometriosis is a chronic gynecological condition characterized by the presence of endometrial-like tissue outside the uterine cavity, affecting almost every third woman at the reproductive age (1). Although is a condition related to autoimmune diseases, the attitude of women influence on the disease and the quality of their lives. Women with a positive attitude felt negative aspects of the disease more rarely (1). The 77.2% of women with endometriosis are symptomatic. This condition leads to significant pain, infertility, and a notable reduction in health-related quality of life (2). The main manifests of this disease would be ovarian endometriosis (OE, also known as endometrioma) and deep infiltrating endometriosis (DE), the latter being the most clinically worrying. OE are cystic masses arising from ectopic endometrial tissue within the ovaries, while DE is characterized by lesions penetrating more than 5 mm beneath the peritoneal surface (3). In USA, the prevalence of OE was 50% in women having irregular menstrual cycle, 30% in women with regular cycle, and 6% in postmenopausal women. In EU, this prevalence was around 7% in pre- and 18% in postmenopausal women (4).

Physiological cysts are most frequent, and minimal treatment is required to resolve it. Surgery may be required if the women have painful, large and persistent cysts (5). In no case is it clear whether minimally invasive surgical interventions improve the quality of life of these women with OE. Data from France indicates that among hospitalizations for OE, approximately 21% of women were discharged without surgery, suggesting a conservative management approach. The remaining 79% underwent surgical intervention (6). The data are limited in Spain. The choice of treatment depends on factors such as the cyst's characteristics and the patient's clinical profile. Surgical techniques include laparoscopically or laparotomy, the laparotomy being the traditional but invasive option, and laparoscopy as a less invasive procedure (7).

Several studies have investigated the impact of endometriosis on quality of life. Pain perception was associated with greater severity, poorer quality of life, and higher levels of anxiety and depression. A review has demonstrated that endometriosis negatively affects all domains of quality of life, being pain and infertility as the major factors (2). OE had also a negative impact on woman's quality of life and most women find difficulties to adapt their life to the disease. For physical health, many women are unable to travel and have less sexual desire. Socially, women reported that their relationship with family and friends are affected, and they needed social support (8). Thus, research indicates that women with OE or DE experience significantly lower physical component compared to controls, highlighting the profound effect of endometriosis subtypes on physical health (3). In addition, although surgical intervention is an option to alleviate pain, the impact of surgery on health-related quality of life remains ongoing research. Some studies suggest that surgery can lead to improvements in certain quality of life domains, while others report persistent impairments postoperatively (9). Notably, a study comparing quality of life in women after DE surgery to standardized Spanish values found that, although there were improvements in bodily pain and mental health, physical and social functions remained low (9).

Despite these insights, there is a paucity of research directly comparing health-related quality of life among women with operated and non-operated OE, those with DE, and women without endometriosis. Thus, the aims of this study were to describe the perception of women with surgery and non-surgery OE in global health and quality of life, to distinguish this profile of women with DE and to compare these variables in healthy women without endometriosis diagnosis.

## 2 Materials and methods

### 2.1 Study design and cohort enrollment

**Figure 1:**
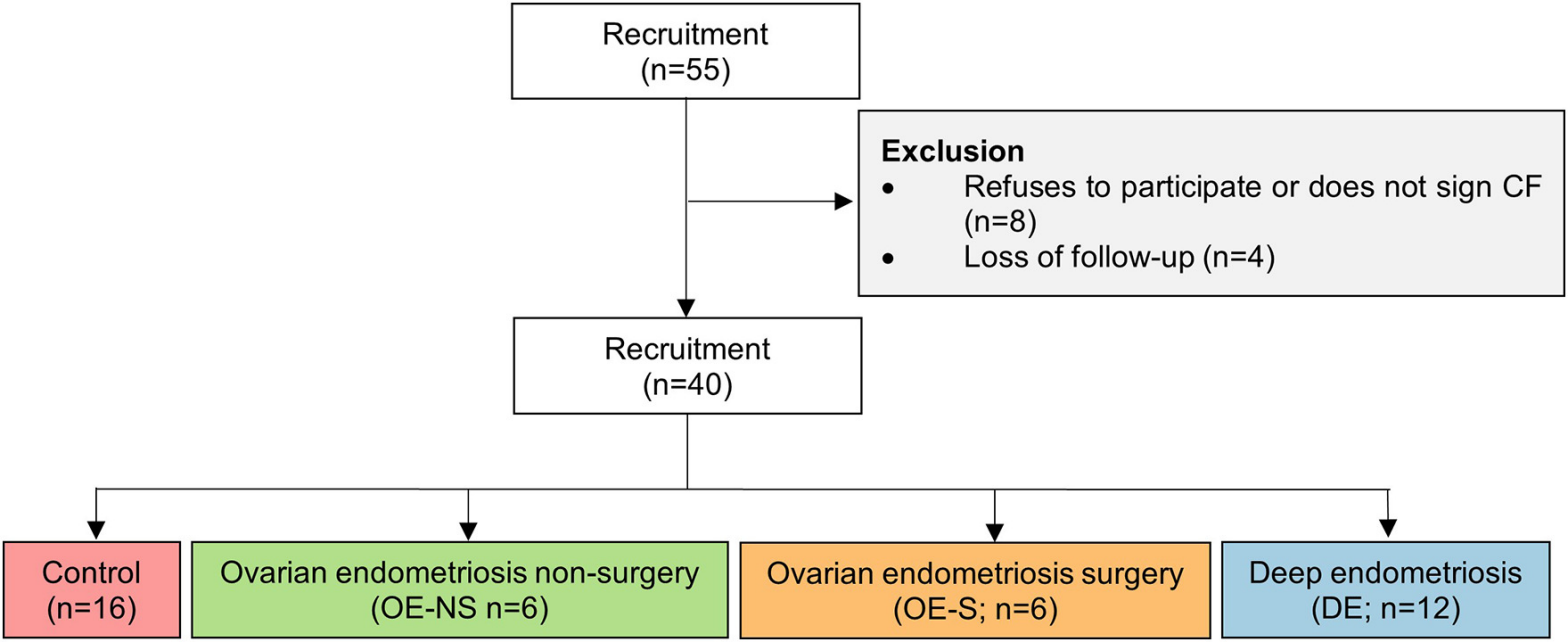
Flow-chart of study enrollment and design. CF, consent form.

This observational study has a cross-sectional design in which women were recruited in the obstetrics and gynecology service within the specialized unit for the follow-up and control of women with endometriosis at Hospital Universitario La Paz (HULP, Madrid, Spain). All women between 25 and 55 years of age, without amenorrhea and associated comorbidities (cancer, mood or emotional disorders, hypertension, obesity) and harmful habits (alcohol consumption or drug abuse) who had been diagnosed with ovarian endometriosis (OE) under pharmacological treatment were invited to participate. These women were categorized according to whether they had undergone surgery (OE-S) or not (OE-NS). In addition, women who had been diagnosed with deep endometriosis (DE) and had undergone ovarian or bowel resection surgery were invited to participate. The women in the control group were recruited from regular gynecological follow-up visits, none of whom had been diagnosed with endometriosis, cancer or had undergone surgery. The recruitment period lasted from March 2023 to October 2024. Finally, a total of 40 women signed the informed consent form and were included in the study (Figure 1). As this was an exploratory study, no sample size calculation techniques were used.

The women were electronically sent a variable collection booklet consisting of sociodemographic questions (*ad-hoc*) including, nationality, educational level, civil status, work situation, monthly income, smoking habits and tobacco index, and following some type of diet. In addition, health and quality of life variables related to endometriosis were collected. The response to the complete booklet did not exceed 25 min. The booklet was handed out at successive clinical follow-up visits.

In addition, the women were scheduled to take a 4 ml blood sample by venipuncture. This sample was used to determine hematological parameters for clinical medicine laboratory of HULP and to test their systemically health at the time of the study.

This design has the approval of the Research Ethical Committee of HULP (PI-5435; approved on 02 December 2022).

### 2.2 Global health variables

#### 2.2.1 Short Form-36 Health Survey (SF-36)

This is a widely used instrument to assess health-related quality of life. Designed by Ware and Sherbourne (10), SF-36 measures perceived health status through 36 items, grouped into 8 dimensions: physical function (10 items), assesses limitations in physical activities due to health problems; physical role (4 items), measures the impact of health on work performance and other daily activities; bodily pain (2 items), assesses the intensity of pain and its interference with daily activities; general health (5 items), reflects the general perception of health status; vitality (4 items), measures energy levels and fatigue; social function (2 items), assesses the impact of health on social life; emotional role (3 items), examines the interference of emotional problems on work and daily activities; and mental health (5 items), assesses symptoms of anxiety, depression, and psychological well-being. Scores for each dimension are transformed into a scale from 0 to 100, where higher values indicate better health. In addition, SF-36 allows for the calculation of 2 global components: the physical component and the mental component. This questionnaire has been validated in various populations and is used in epidemiological and clinical studies (11, 12). In addition, SF-36 was applied in women with endometriosis (13, 14) and has showed association with visual analogue scale of pain in women with endometriosis and surgery (15).

#### 2.2.2 Pain Catastrophizing Scale (PCS)

This is an instrument designed to assess the tendency to catastrophize pain (16), a psychological factor that can influence perception and coping with chronic pain. PCS has been widely used in studies on pain, including pain associated with endometriosis, where it has been linked to higher levels of disability and emotional suffering (17). The scale consists of 13 items, which assess three dimensions: rumination (4 items), repetitive thoughts about pain and difficulty distracting oneself, magnification (3 items), exaggerated perception of pain and its consequences and helplessness (6 items), feeling of inability to control or relieve pain. Each item is rated on a Likert-type scale from 0 (never) to 4 (always), with a total score ranging from 0 to 52, where higher values indicate greater pain catastrophizing. PCS is a useful tool in the psychological assessment of women with endometriosis and in the design of interventions to improve their well-being.

#### 2.2.3 Postpartum Depression Predictors Inventory-Revised (PDPI-R)

Originally, PDPI-R was an instrument designed to assess postpartum depression (18). However, one of the subscales can assess the perceived practical, social and emotional support by 12 items, including 4 for partner, 4 for family, and 4 for friend's support (no = 0, yes = 1; range 0–12). This Spanish version was previously validated and used (19). High scores mean high perceived social support. In our cohort, the internal consistency was 0.80.

### 2.3 Quality of life variables with endometriosis

#### 2.3.1 Endometriosis Health Profile-5 (EPH-5)

This is a shortened version of the Endometriosis Health Profile-30 questionnaire, designed to assess the quality of life in women with endometriosis (20). Its development was based on the need for a brief and effective tool to measure the impact of the disease in various areas of daily life (21). EPH-5 consists of 11 items that address the most representative dimensions of endometriosis symptomatology, five items including intensity and frequency of pain, impact of the disease on emotional stability and mood, the impact on social interactions, and self-image from the core questionnaire and six items from the modular questionnaire that may not be applicable to every woman with endometriosis including work, intercourse, and worries about infertility, treatment, and relationship with children and medical professionals. Each item is rated on a Likert scale (never=0 to always=4 and not relevant if not applicable). Scores on EHP-5 core and modular questionnaire then are transformed on a scale of 0 (indicating best possible health status) to 100 (indicating worst possible health status). If the “not relevant” box was ticked for items on modular questionnaire the score could not be computed for that dimension. EPH-5 shown an internal consistency of 0.71 (22) and has been used in Spanish population (23).

#### 2.3.2 Stellenbosch Endometriosis Quality of Life (SEQOL)

SEQOL is a validated questionnaire designed to assess the quality of life in women with endometriosis. SEQOL provides a comprehensive evaluation of the physical, emotional, and social impact of the disease (24). The version comprises 35 items clustered in key domains such as psychological well-being (7 items), support (3 items), vitality (3 items), sexual and relationship function (5 items), occupational function (4 items), reproductive function (6 items), menstrual characteristics (3 items) and failures related to life benefits (4 items). Each item is rated on a Likert scale (never = 5 to very = 1) with a total score ranging from 1 to 5, where higher values indicate greater life functionality. SEQOL has demonstrated reliability of 0.92 (25), making it a valuable instrument for clinical research and patient-centered care in endometriosis management.

### 2.4 Statistical analysis

The analysis was performed by R software version 4.4.1 (R Core Team 2022, Vienna, Austria; https://www.R-project.org/) with the RStudio interface (version 2023.06.0 + 421 for Windows; Boston, MA, USA). The packages used were *rio, dplyr, compareGroups, tidyverse, ggplot2, ggpubr, patchwork, corrplot*, and *pheatmap*.

Considering the sample size of the study, robust nonparametric techniques were used to avoid bias from atypical data. The data was summarized by sample size (n) and relative frequency (%) in the categorical variables and median and interquartile range [Q1; Q3] in the quantitative variables. The contrast by groups was performed by Fisheŕs exact test in the categorical variables and Kruskal-Walli's test in the quantitative variables. The *post-hoc* analysis was verified by Tukeýs Honestly significant differences (HSD) test. Effect size (Cohen's d) was calculated to estimate the magnitude (absolute value) of significant group differences (d ≥ |0.8| indicate large effect, being substantially important). These metrics were included to enhance the interpretability of the findings. The correlations between global health and quality of life variables were tested by Spearman's Rho coefficient. In addition, heatmap analysis was performed to cluster the groups by similarities in global health and quality of life using Euclidean distance between variables. This visual approach allows an intuitive understanding of the relationships among the groups based on their psychological profiles, highlighting the distinct clusters within the data. Therefore, the data were typed by min and max value. Additionally, fold-change was represented by linear regression model (LRM). To calculate the fold-change for each quality of life variable, the median of OE or DE group was subtracted to the median of control group. This difference was divided by median value of control, and then, the fold-change values were plotted. For LRM, each quality of life dependent variable was included and the endometriosis group (OE or DE) served as the independent variable. The control group was consistently used as the reference, allowing for comparisons between each endometriosis group and control (i.e., OE vs. control, DE vs. control). From LRM, the *P*-value (*P*) of the standardized coefficient was extracted to classify fold-change as significant. No missing data imputation techniques were used in this study. The *P* < 0.05 was considered statistically significant in all analyses.

## 3 Results

### 3.1 Social context and hematological parameters

The women had 36.0 [30.0; 44.0] years old, being Spanish the 91.9%. There were no differences between groups in the social variables. No significant differences were detected between groups in hematological and coagulation parameters (Table 1).

**Table 1:** Sociodemographic and blood parameters between groups of women with endometriosis.

Variables	Control (*n* = 14)	OE-NS (*n* = 5)	OE-S (*n* = 6)	DE (*n* = 10)	*P*
Age (year)	33.0 [28.5; 36.8]	30.0 [27.0; 38.0]	38.5 [36.2; 43.8]	42.0 [34.2; 44.0]	0.159
Spanish	13 (92.9%)	5 (100%)	4 (80.0%)	9 (90.0%)	0.848
Education					0.101
High school	0 (0.0%)	1 (20.0%)	1 (20.0%)	3 (30.0%)	
University	14 (100%)	4 (80.0%)	4 (80.0%)	7 (70.0%)	
Civil status					0.948
Single	4 (28.6%)	2 (40.0%)	3 (50.0%)	4 (44.4%)	
With couple	6 (42.9%)	1 (20.0%)	2 (33.3%)	3 (33.3%)	
Married	4 (28.6%)	2 (40.0%)	1 (16.7%)	2 (22.2%)	
Employee	14 (100%)	4 (80.0%)	6 (100%)	10 (100%)	0.143
Monthly income					0.779
1,001–2,500€	5 (35.7%)	3 (60.0%)	4 (66.7%)	6 (60.0%)	
2,501–4,000€	3 (21.4%)	0 (0.0%)	0 (0.0%)	2 (20.0%)	
>4,000€	5 (35.7%)	1 (20.0%)	1 (16.7%)	1 (10.0%)	
Smoker	2 (14.3%)	1 (20.0%)	4 (66.7%)	2 (20.0%)	0.120
Tobacco index	0.5 [0.5; 0.5]	21.0 [21.0; 21.0]	13.8 [9.8; 15.8]	2.4 [2.4; 2.4]	0.248
Follow some type of diet	2 (14.3%)	2 (40.0%)	0 (0.0%)	4 (40.0%)	0.189
Erythrocytes (×10^6^/µl)	4.5 [4.4; 4.6]	4.7 [4.5; 4.9]	4.6 [4.5; 4.8]	4.7 [4.5; 4.9]	0.290
Hemoglobin (g/dl)	13.5 [13.2; 14.3]	14.4 [14.1; 15.1]	14.0 [13.5; 14.4]	14.1 [13.7; 14.6]	0.239
Hematocrit (%)	41.9 [40.8; 44.3]	45.0 [42.8; 46.7]	43.6 [42.8; 44.2]	44.0 [42.5; 44.8]	0.405
MCV (fl)	94.3 [90.6; 97.4]	94.2 [93.5; 95.0]	92.9 [91.2; 96.0]	91.3 [90.2; 95.5]	0.889
MCH (pg)	30.8 [29.4; 31.6]	31.0 [30.9; 31.3]	29.5 [28.8; 31.4]	30.4 [29.4; 30.9]	0.383
Platelets (×10^3^/µl)	246 [214; 258]	234 [204; 292]	262 [232; 278]	259 [218; 290]	0.547
MPV (fl)	8.1 [7.8; 8.6]	8.4 [8.0; 9.5]	8.0 [7.7; 8.4]	7.7 [7.4; 8.4]	0.563
PAT (seg)	11.3 [11.1; 11.5]	11.1 [10.8; 11.6]	10.9 [10.6; 11.1]	10.9 [10.6; 11.1]	0.119
Prothrombin activity (%)	102 [100; 112]	106 [94.5; 114]	113 [106;118]	110 [107; 118]	0.239
Fibrinogen (mg/dl)	279 [258; 319]	310 [298; 324]	311 [286; 343]	332 [294; 428]	0.178
TAT (seg)	26.4 [25.9; 27.3]	29.3 [27.8; 29.8]	26.8 [26.0; 28.4]	26.9 [25.8; 29.0]	0.239
PTAT (a.u.)	0.99 [0.97; 1.02]	1.08 [1.03; 1.10]	0.99 [0.96; 1.05]	1.00 [0.96; 1.06]	0.302

Data shows median and interquartile range in the quantitative variables and sample size (*n*) and relative frequency (%) in the categorical variables. The *P*-value (*P*) was extracted from Kruskal-Walli's test in the quantitative variables and Fisheŕs exact test in the categorical variables. The groups with different letters indicate *P* < 0.05 by Tukey HSD test. OE, ovarian endometriosis; NS, non-surgery; S, surgery; DE, deep endometriosis; MCV, mean corpuscular volume; MCH, mean corpuscular hemoglobin; MPV, mean platelet volume; PAT, prothrombin activation time; TAT, thromboplastin activation time; PTAT, partial thromboplastin activation time.

### 3.2 Global health profile

**Figure 2:**
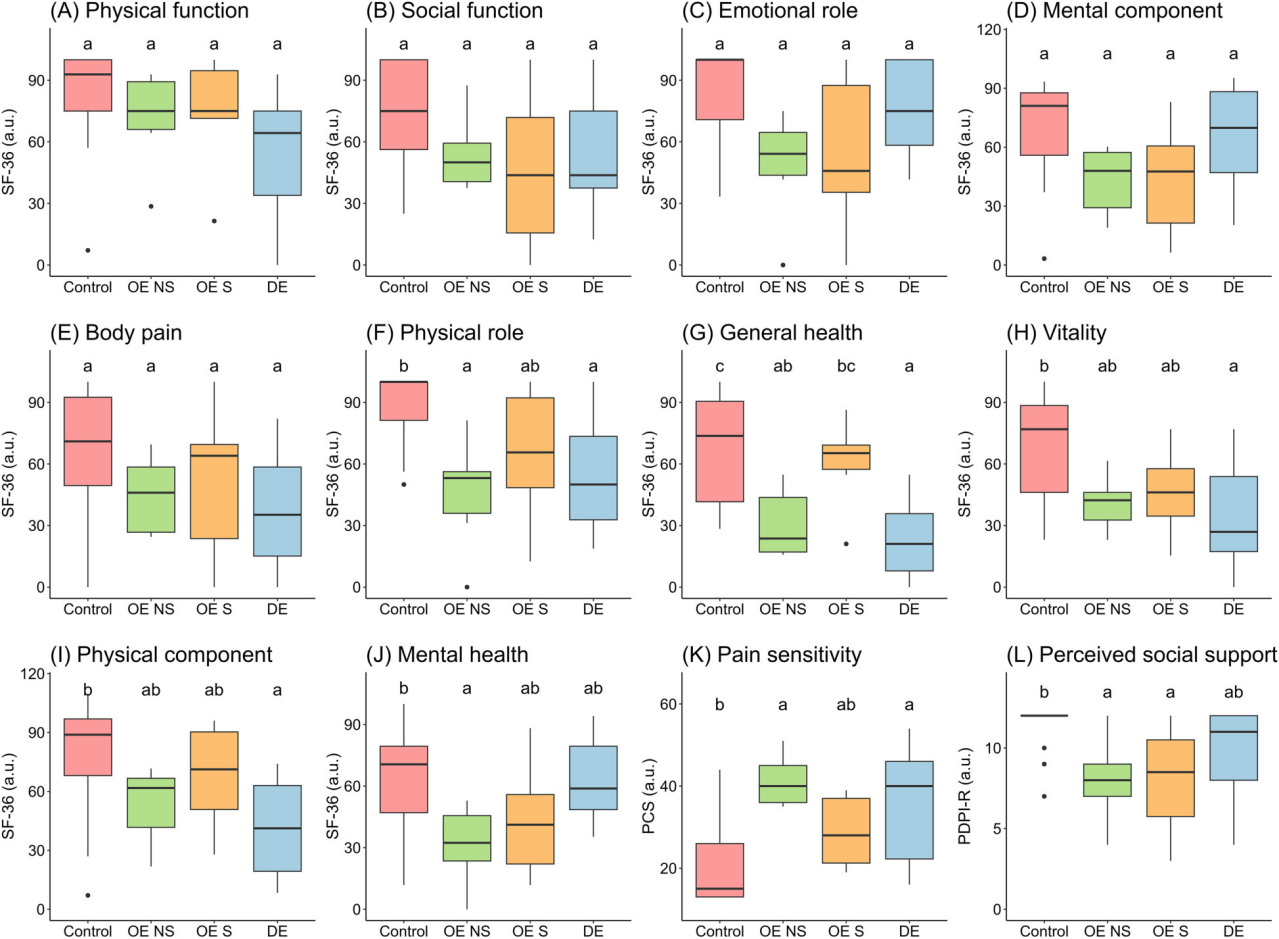
Global health spheres in women by endometriosis groups. Data shows median and interquartile range. The groups with different letters indicate *P* < 0.05 by Tukey HSD test. OE, ovarian endometriosis; NS, non-surgery; S, surgery; DE, deep endometriosis; SF-36, Short Form-36 Health Survey; PCS, Pain Catastrophizing Scale; PDPI-R, subscale of support from Postpartum Depression Predictors Inventory-Revised.

Physical and social functions, emotional role, body pain and global mental component did not show statical differences between groups (Figures 2A–E). Physical role was significantly lower in OE-NS (*d* = 1.73) and DE (*d* = 1.36) compared to control women; OE-S group showed no significant differences with the control group (Figure 2F). General health was significantly lower in OE-NS (*d* = 1.65) and DE (*d* = 1.89) groups compared to the control. OE-S showed higher score than DE with no significance compared to control (Figure 2G). Vitality (*d* = 1.25) and physical component (*d* = 1.53) were significantly lower in DE compared to control. Women with OE showed intermediate scores between DE and control groups (Figures 2H,I). Mental health was significantly lower in women with OE-NS compared to control (*d* = 1.37), with no alteration in the other groups (Figure 2J). Pain sensitivity was higher in OE-NS (*d* = 2.43) and DE (*d* = 1.18) compared to control and women with OE-S did no show differences with control (Figure 2K). The perception of social support was lower in OE compared to control, with non-differences with DE (Figure 2L).

### 3.3 Quality of life profile

**Figure 3:**
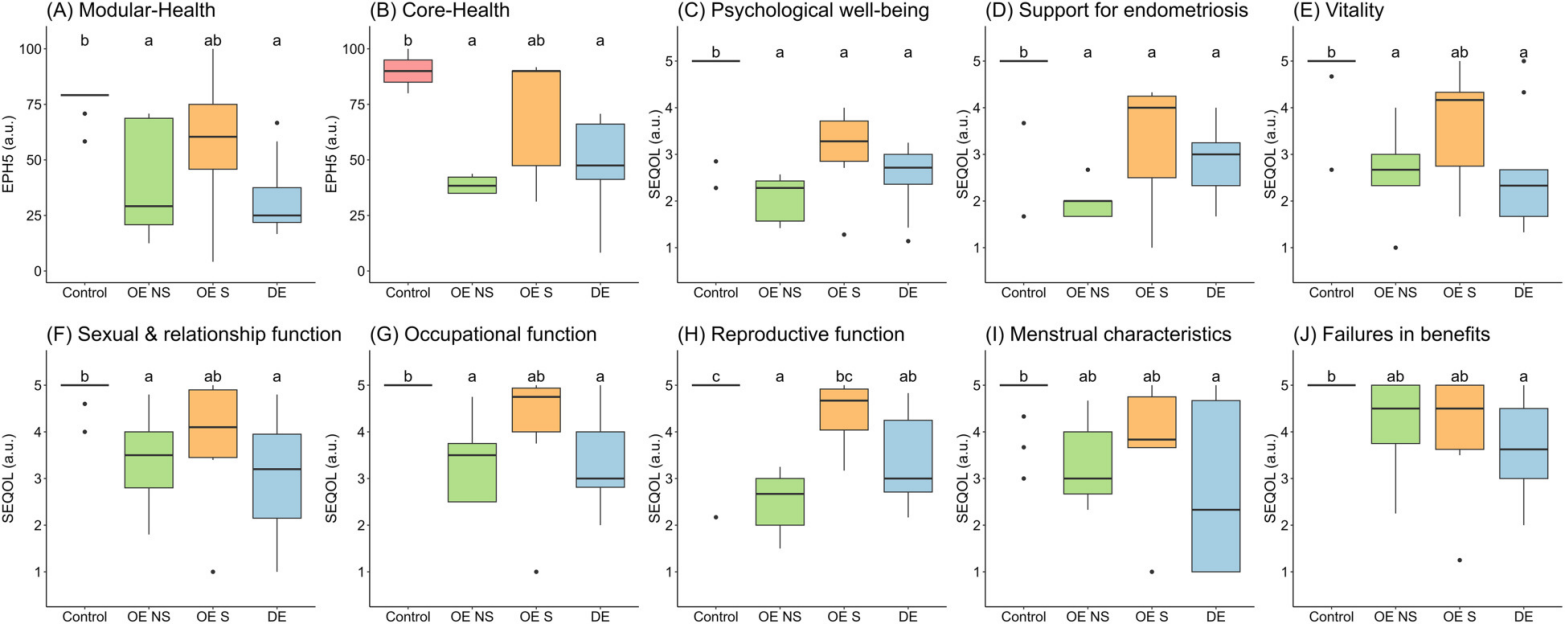
Quality of life profile with endometriosis between groups of women. Data shows median and interquartile range. The groups with different letters indicate *P* < 0.05 by Tukey HSD test. OE, ovarian endometriosis; NS, non-surgery; S, surgery; DE, deep endometriosis; EPH-5, Endometriosis Health Profile-5; SEQOL, Stellenbosch Endometriosis Quality of Life.

The core and modular health profile were significantly lower in DE and OE-NS groups compared to control. OE-S did not show significant differences with DE or control groups (Figures 3A,B). Psychological well-being and support for endometriosis were significantly lower in all groups of women with endometriosis compared to control (Figures 3C,D). Similar to control, vitality, sexual and occupational functions were higher in OE-S compared to OE-NS and DE women (Figures 3E–G). Reproductive function was significantly lower in OE-NS compared to OE-S (*d* = 2.61) and control (*d* = 3.13) (Figure 3H). The menstrual characteristics of women with endometriosis were significantly lower compared to control, but did not show differences with OE women (Figure 3I). Finally, the perception of failures in life benefits was significantly lower in DE compared to control (*d* = 1.89), while OE groups showed no significant differences (Figure 3J).

### 3.4 Relationship between global health and quality of life profile

**Figure 4:**
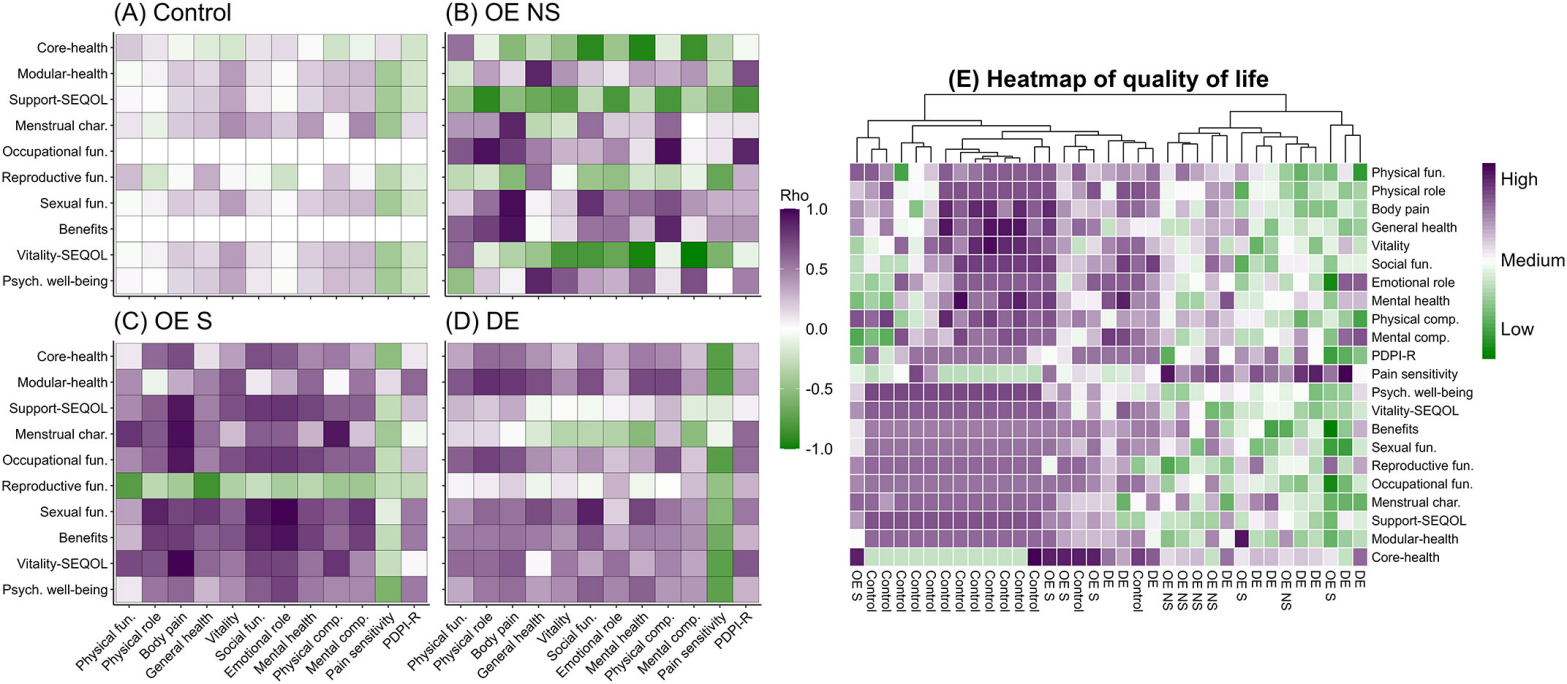
Correlograms and heatmap of global health and quality of life between women with endometriosis. In correlograms, data sown Spearman's Rho coefficient. In heatmap, the value shown typified data of the variable. fun, function; Psych, psychological; OE, ovarian endometriosis; NS, non-surgery; S, surgery; DE, deep endometriosis, subscale support from Postpartum Depression Predictors Inventory-Revised (PDPI-R); Stellenbosch Endometriosis Quality of Life (SEQOL).

The correlation matrices showed that OE-S had intermediate correlations patterns between control and women with DE, while women with OE-NS presented similar correlations to DE group (Figure 4). The heatmap between quality of life and health profile variables showed that women in OE-S group were close to control while OE-NS was close to women with DE (Figure 4E).

**Figure 5:**
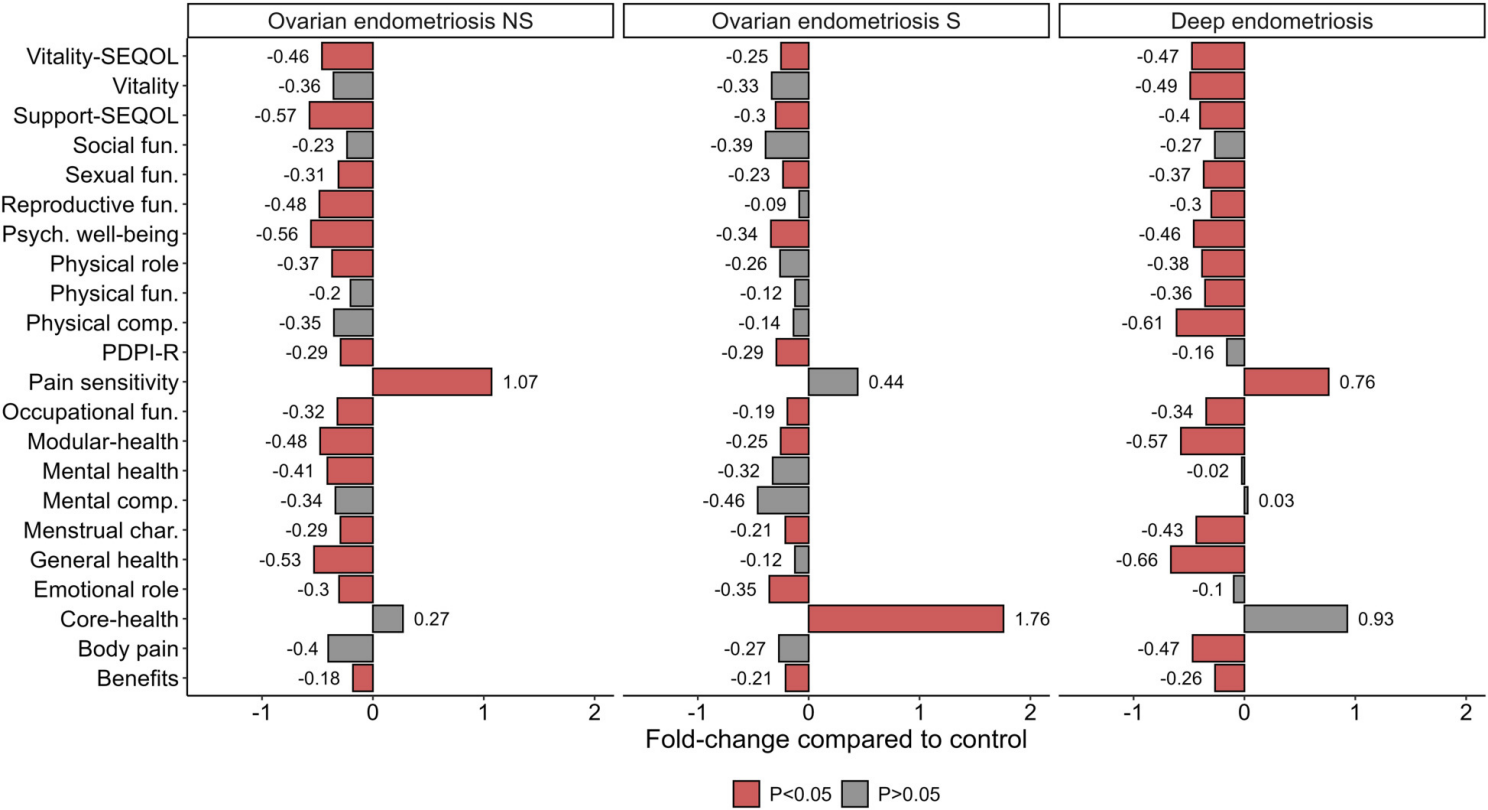
Fold-change of health and quality of life profile by women with endometriosis. Data shows fold-change from control group. The *P*-value (*P*) was extracted from linear regression model. fun, function; Psych, psychological; NS, non-surgery; S, surgery; PDPI-Rsubscale support from Postpartum Depression Predictors Inventory-Revised; SEQOL, Stellenbosch Endometriosis Quality of Life.

Quality of life variables showed negative fold-change in women with endometriosis compared to control. Exceptionally, women with endometriosis had higher fold-change than control in pain sensitivity and core module of health, being significantly different for pain in OE-NS and DE and in core health for OE-S. Compared to control, women with OE-NS had 15 significantly altered quality of life variables, while women with OE-S had 11. Women with DE had 16 modified variables (Figure 5).

## 4 Discussion

**Figure 6:**
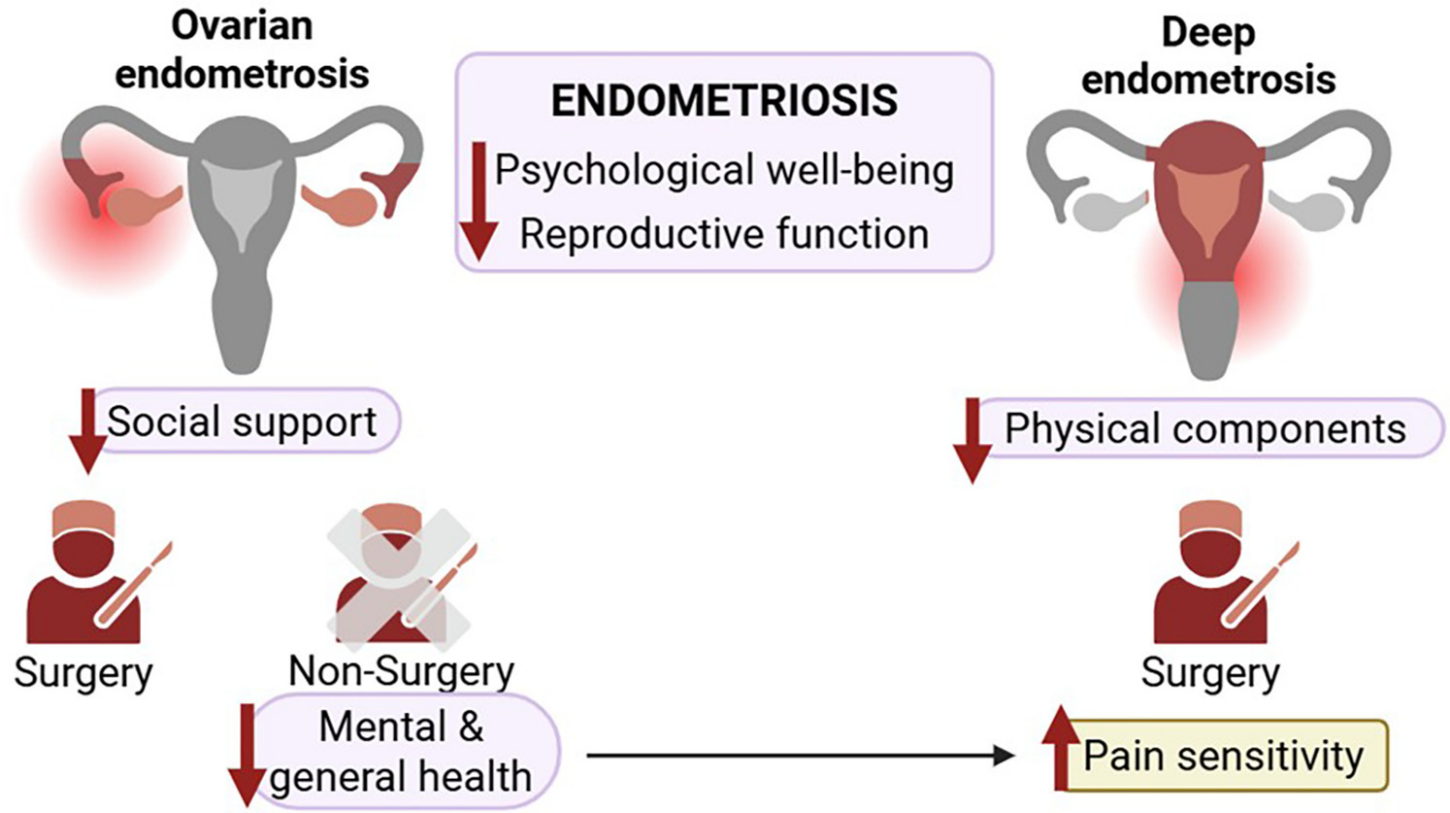
Differential impact of ovarian and deep endometriosis on quality of life domains and surgery outcomes. Endometriosis is associated with reduced psychological well-being and reproductive function. In ovarian endometriosis, decreased social support was observed. Women with ovarian endometriosis who do not undergo surgery report poorer mental and general health. Women with deep endometriosis exhibit decline in physical components and increased pain sensitivity.

This study examined the quality of life and health profiles of women with different types of endometrioses compared to women without this disorder. Women with DE and OE-NS had low physical role functioning and general health. Physical components were also reduced in DE, while mental health was lower in OE-NS. Pain sensitivity was high in DE and OE-NS, whereas social support perception was reduced in ovarian endometriosis. Psychological well-being, reproductive function, and endometriosis-specific support were impaired in endometriosis groups (Figure 6). In addition, OE-NS group closely resembled DE, while OE-S was closer to control. Overall, women with endometriosis exhibited a worsened quality of life, showing OE-NS the most meaningful alterations.

Women with ovarian endometriosis experience depression and anxiety due to pain and delayed diagnosis (26). The present data show that mental health in OE-NS was −0.41 fold-change respect to control, but in OE-S was non-significant −0.32 and in DE similar to control, possibly show coping to the disease. Women who have accepted their disease and have learned to live with it better tolerate pain and cope with other symptoms, and they report a higher level of the quality of life, despite the presence of the disease (27). Understanding by family members and partners may help women cope with this disease. In an observational study it was shown that satisfying social support was the key resources for resilience in endometriosis (28). Furthermore, in women with endometriosis was showed that social loneliness was similar to general population, but emotional loneliness was greater, suggesting a lack of intimacy in close relationships (29, 30). According to our data, the perception of support was low in all women with endometriosis. However, women with DE had slightly better scores, which may indicate a longer disease process with longer acceptance time.

Self-esteem is affected in 67% of the cases and 59% report impaired functioning (31). According to our data, the physical function was similar in all women, but sexual, reproduction and occupational functions were deeply decreased in OE-NS, being restored in OE-S. In women with ovarian endometriosis, social life is also impacted with 56% missing events due to pain. Women report a greater number of sexual contacts, a high level of satisfaction, improved relationships with their partners, high self-esteem, and a reduced number of depressive episodes (1). In our data, although social function was not altered, vitality and pain sensitivity could be affected, being high in OE-NS and DE, however in OE-S was similar to control. Many women with DE feel like a burden, fear job loss due to frequent absences, and feel helpless about their future. Many of them have missed work due to the condition (32). Other conditions described as affecting women with endometriosis include sleep disturbances with high rate experiencing issues every night (33, 34). Causes that can lead to fatigue and worsen the overall well-being health profile of these women. In our data, the psychological well-being was in a range 0.34–0.56 lower compared to control.

Our findings align with previous studies indicating that endometriosis is associated with significantly reduced quality of life (35). Increased pain sensitivity in DE and OE-NS may be linked to inflammatory and neuropathic mechanisms previously described (36). The low social support perception among women with endometriosis highlights the need for comprehensive psychosocial interventions. Women cope with the disease trying nonconventional methods, such as herbal therapies, physical activity, or psychotherapy. A qualitative study revealed that all women improve their quality of life with cognitive behavioral therapy, which should be added to the standard treatment (37). Women should be informed about the negative effects of ovarian endometriosis on their quality of life, while health professionals should recognize and assess patients' symptom experiences, their impact on daily life, and individual care priorities to enhance treatment outcomes (8).

Both pharmacological and surgical treatment improves the quality of life in women suffering endometriosis (38, 39). In pharmacological treatment, hormonal therapy is used to reduce of pain. Improvement in well-being and the quality of sexual life was also observed in patients treated with surgery. Laparoscopic surgery has been shown to improve sexual and overall health-related quality of life in women with DE (40), increasing quality of life (41). A Cochrane review concluded that excision surgery for endometriomas provides better outcomes than drainage and ablation in terms of cyst recurrence, pain symptoms, and spontaneous pregnancy rates in previously infertile women (42). In addition, these effects are additionally enhanced by pharmacotherapy after surgery (43).

### 4.1 Clinical implications and implemented protocols

Treatment planning should consider endometriosis subtype and severity, as different presentations may require specific therapeutic approaches. The reduced social support perception in women with endometriosis highlights the need to provide psychological resources and support groups to improve overall well-being. Interventions should incorporate regular assessments for psychological distress and referrals to suitable mental health and social support services. In addition, a structured psychological intervention plan can enhance quality of life and help women with ovarian cysts cope with physical and emotional challenges before and after surgery. Key components include: (1) preoperative support with counseling, education, and relaxation techniques to reduce anxiety; (2) cognitive-behavioral therapy to address negative thoughts, develop coping strategies, and improve problem-solving; (3) pain management training using biofeedback and relaxation techniques; (4) social and emotional support through peer groups and family counseling; and (5) postoperative recovery support with reintegration strategies, stress management, and follow-up psychological care. Given the limited resources available to address psychological distress in this population, women should receive care in a specialist center (44). Routine distress screening and timely referrals to psychological and social services can help enhance the quality of endometriosis life. In addition, given the sensitivity of discussing reproductive health, especially among women with endometriosis, women should be given the opportunity to discuss the emotional distress it causes them. Provide supportive social and health care environment for women.

### 4.2 Limitations and futures directions

While this study provides valuable insights, several limitations should be acknowledged. One of them would be the relatively small sample size. Additionally, the uneven distribution of participants across groups could introduce bias. These factors may also constrain the generalizability of the findings to broader populations. The limited and unbalanced sample was due to contextual challenge inherent to the recruitment process, particularly given the clinical nature of the population studied. Furthermore, it should be noted that surgery could also have a negative impact on quality of life, especially in the immediate after it. Although this has not been considered in this study, it would be highly relevant to explore this aspect in future research. Therefore, longitudinal studies should explore mechanisms underlying quality of life differences across endometriosis subtypes and evaluate targeted interventions and effect of surgical processes. Additionally, due to the cross-sectional and observational design of this study, causal relationships cannot be established, limiting the interpretation of associations found. Additionally, it would be important to consider recording, and therefore modulation, of fold-change for other factors, such as comorbidities, since endometriosis can coexist with other health issues that affect women's quality of life.

## 5 Conclusion

Women with non-surgically treated ovarian endometriosis exhibit a lower global health profile compared to women without endometriosis, whereas surgically treated ovarian endometriosis shows no differences with control group. Psychological well-being and perceived social support are lower in women with endometriosis, highlighting a common impact of the disease on mental health. Notably, vitality, sexual, and occupational functions are better preserved in surgically treated ovarian endometriosis than non-surgically treated. Reproductive function is the most compromised in non-surgically ovarian endometriosis treated but remains similar between surgically treated and controls, being menstrual characteristics similar affected in all endometriosis groups. This approach should be achieved by a multidisciplinary team of gynecologists, psychologists, or even nutritionists. However, although they may be more adaptable to the disease, quality of life in women with deep endometriosis remains a challenge to be addressed.

## Data Availability

The original contributions presented in the study are included in the article/Supplementary Material, further inquiries can be directed to the corresponding author.
